# Peer Review of Grant Applications: A Simple Method to Identify Proposals with Discordant Reviews

**DOI:** 10.1371/journal.pone.0027557

**Published:** 2011-11-14

**Authors:** Bruno Giraudeau, Clémence Leyrat, Amélie Le Gouge, Julie Léger, Agnès Caille

**Affiliations:** 1 Institut National de la Santé et de la Recherche Médicale, Tours, France; 2 Université François Rabelais, Tours, France; 3 Centre Hospitalier Régional et Universitaire de Tours, Tours, France; 4 Institut National de la Santé et de la Recherche Médicale, Paris, France; Rutgers University, United States of America

## Abstract

Grant proposals submitted for funding are usually selected by a peer-review rating process. Some proposals may result in discordant peer-review ratings and therefore require discussion by the selection committee members. The issue is which peer-review ratings are considered as discordant. We propose a simple method to identify such proposals. Our approach is based on the intraclass correlation coefficient, which is usually used in assessing agreement in studies with continuous ratings.

## Introduction

Peer review is now the principal mechanism for selecting grant applications for funding [1], [2]. In this process, inter-reviewer agreement is important for ease in application ranking. Both Wiener *et al* [3] and Hartmann *et al* [4] found high inter-reviewer agreement in rating proposals. Green *et al* [5] demonstrated that the rating intervals of the scale (0.5 or 0.1) did not influence the final assessment. Nevertheless, reviewers still have disagreements about some proposals because of differing scientific backgrounds, perceptions of the proposal, or non-declared conflicts of interest. Proposals with discordant peer-review ratings need to be discussed before a global ranking of proposals. We propose a simple method to help selection committees identify proposals that require discussion because of lack of agreement in peer-reviews.

### Example

Let us consider the example of 20 proposals submitted to a fictitious funder and assessed by 3 reviewers. Ratings are displayed in Table 1, and, for each proposal we have estimated the intra-proposal mean rating and standard deviation. Disagreement among ratings translates into a high intra-proposal standard deviation for proposals 3, 14, 19, 20 and 15, for example.

**Table 1:** Fictitious example of a number of proposals submitted for funding and rated by 3 raters for application of the formula by Giraudeau *et al.* [9] to identify proposals with discordant peer-review ratings (see appendices).

Proposal no.	Proposal ratings			Intra-proposal rating		Formula		Re-estimated ICC(*)
	Rater 1	Rater 2	Rater 3	Mean	SD	First term	Second term	
1	15.0	13.3	16.7	15.0	1.7	0.0018	−0.0095	0.374
2	11.7	11.7	10.0	11.1	1.0	0.0876	−0.0036	0.282
3	10.0	13.3	18.3	13.9	4.2	0.0134	−0.0618	0.415
4	16.7	16.7	MD	16.7	0	0.0038	0	0.363
5	18.3	13.3	18.3	16.6	2.9	0.0035	−0.0282	0.391
6	20.0	15.0	16.7	17.2	2.5	0.0091	−0.0218	0.379
7	16.7	16.7	18.3	17.2	0.9	0.0089	−0.0028	0.360
8	11.7	11.7	13.3	12.2	0.9	0.0476	−0.0030	0.322
9	20.0	20.0	18.3	19.4	1.0	0.0564	−0.0034	0.313
10	18.3	13.3	18.3	16.6	2.9	0.0034	−0.0280	0.391
11	16.7	18.3	18.3	17.8	0.9	0.0163	−0.0028	0.353
12	10.0	13.3	11.7	11.7	1.7	0.0668	−0.0099	0.309
13	18.3	20.0	16.7	18.3	1.7	0.0271	−0.0093	0.349
14	20.0	13.3	18.3	17.2	3.5	0.0089	−0.0417	0.399
15	20.0	13.3	16.7	16.7	3.4	0.0037	−0.0382	0.401
16	16.7	18.3	13.3	16.1	2.6	0.0006	−0.0217	0.387
17	20.0	16.7	16.7	17.8	1.9	0.0170	−0.0122	0.362
18	13.3	16.7	13.3	14.4	2.0	0.0060	−0.0128	0.373
19	10.0	15.0	16.7	13.9	3.5	0.0127	−0.0420	0.396
20	15.0	16.7	10.0	13.9	3.5	0.0127	−0.0420	0.396

MD: missing data - SD: standard deviation - ICC: intraclass correlation coefficient.

The global mean equals 15.7. The inter-proposal SD equals 2.3.

(*)Baseline ICC is estimated at 0.366, considering the whole sample. The last column of the table displays re-estimated ICCs using Giraudeau *et al*. formula once a proposal is discarded. As an example, for proposal 1, the ICC is derived as 0.366 – (0.0018–0.0095), which equals 0.374.

### A simplistic approach

A simple way to identify proposals with discordant peer-review ratings would be to specify a ceiling intra-proposal standard deviation: each proposal with an intra-proposal standard deviation greater than this ceiling value would be considered as having discordant peer-review ratings. Nevertheless, such an approach would have 2 limits. First, this ceiling standard deviation would highly depend on the rating scale (and would therefore differ for each funder). Second, the ceiling standard deviation should be fixed relative to the inter-proposal heterogeneity rather than be an absolute value. Thus, in our example, if we consider the proposal rating means (i.e., the series 15.0, 11.1 … 13.9 in Table 1), the inter-proposal standard deviation is estimated at 2.3. Then, an intra-proposal standard deviation of 3 or 4 would be unacceptably high but would not be high had the estimated inter-proposal standard deviation been around 5.

### Underlying concept of the proposed approach

Considering that the underlying question of our research is agreement, we focus on the intraclass correlation coefficient (ICC), the parameter usually assessed for continuous outcomes [6]. This coefficient is defined as the ratio of the inter-subject variance (here the inter-proposal variance) to the whole variance (here the inter-proposal variance plus the intra-proposal variance). Thus, the ICC theoretically varies between 0 and 1 [7], where 0 is total lack of agreement among ratings and 1 is perfect agreement with no intra-proposal variance. In our example the ICC is estimated at 0.366 (using the ANOVA estimator in absence of an explicit maximum likelihood estimator when the number of ratings per proposal varies [8]), which can be interpreted as 36.6% of the total variation being due to inter-proposal variability (i.e., the “true” variability) and 63.4% to lack of agreement among reviewers.

Giraudeau *et al.* [9] derived an analytical formula that assesses the influence of a subject (here, a proposal) on the estimate of the ICC (Appendix S1). For a given proposal (named *i_0_* for convenience), this influence is actually the sum of 2 antagonist effects: the positive effect, related to the *i_0_* mean rating (the ICC would be high with a very low [or very high] mean rating for a proposal) and a negative effect, related to the variance of the *i_0_* ratings (the ICC would be low with high heterogeneity of ratings). Giraudeau *et al* developed an explicit formula in the balanced case (i.e., with a common fixed number of ratings per proposal), but this formula still approximates accurately the influence of a proposal in the unbalanced case (i.e., when the number of peer-review ratings varies among proposals) (Appendix S2). In our example, if we focus on proposal 3, the first term (effect) is estimated as 0.0134 and the second term −0.0618 (Table 1). Because this proposal has a mean rating not very different from the global mean (i.e., 13.9 *vs* 15.7), the first term is small. In contrast, because of disagreement in ratings for this proposal, its intra-rating standard deviation is estimated as 4.2 and the second term is high, in absolute value. If this proposal were to be discarded from the sample, the re-estimated ICC would be 0.415 which is derived from 0.366 (the whole sample ICC estimate) minus 0.0134 (the positive effect of the mean ratings) minus −0.0618 (the negative effect of the intra-proposal standard deviation).

## Results

### Proposed approach

We then propose to use the second term of the formula to identify proposals with discordant reviews by the following algorithm:

1. Discard any proposal with only one rating, considering that it automatically needs to be discussed.
2. Estimate the ICC for the thus truncated dataset.
3. Apply the analytical formula for each proposal.
4. Identify the proposal for which the second term of the formula is highest in absolute value (i.e., the proposal that has the greater negative impact on the ICC estimate).
5. Discard the identified proposal from the sample. In case of ties, discard all proposals for which the second term of the formula is equally high (in absolute value).
6. Estimate the ICC for the truncated sample.
7. Repeat steps 3 to 7 until the ICC estimate has reached a pre-specified value.
8. The discarded proposals are those that need to be discussed because of peer-review rating disagreement.

The code to implement this algorithm is presented in Appendix S3.

In this algorithm, the only arbitrary choice is the ceiling ICC required in step 7, which must be pre-specified for the following reason: specifying this 0.7 value, for instance, means that in the final sample (i.e., once all proposals with too-high discordant ratings have been discarded), 70% of the variability is due to “true variability” (i.e., variability among proposals) and 30% is due to inter-reviewer heterogeneity (i.e., variability within proposals). We consider this reasoning more concrete and easier than specifying a ceiling intra-proposal standard deviation because the ceiling ICC value is independent of the rating scale and the funder requirements.

### Example

We applied this algorithm to the dataset previously presented using a threshold value of 0.7 for the ICC. Seven proposals were identified as needing discussion because of disagreements among ratings (Table 2). The first proposal discarded is proposal 3, which results in a sample of 19 proposals and an estimated ICC of 0.415. The second proposal discarded is 19, which results in an estimated ICC of 0.449; the third proposal is 20 etc. Once proposals 3, 19, 20, 14, 15, 5 and 10 have been discarded, the estimated ICC is 0.708. Obviously, a cut-off value of 0.7 for the ICC is a stringent constraint and leads to a high number of proposals needing discussion (7 of 20). We may decide to be less stringent; for instance, an ICC of 0.5 (i.e., 50% of the variability is due to “true variability”) would lead to identifying only 4 proposals (proposals 3, 19, 20 and 14).

**Table 2:** Process of identifying proposals with discordant peer-review ratings from the dataset in Table 1.

	Discarded proposals	ICC	Proposal to be discarded for the next round
Baseline		0.366	3
Round 1	3,	0.415	19, 20
Round 2	3, 19, 20	0.487	14
Round 3	3, 14, 19, 20	0.542	15
Round 4	3, 14, 15, 19, 20	0.603	5, 10
Round 5	3, 5, 10, 14, 15, 19, 20	0.708	STOP(*)

(*)At round 5, the ICC estimate was greater than the 0.7 ceiling, so the algorithm was stopped.

## Discussion

We propose a simple way to identify proposals for which inter-reviewer ratings are discordant. Obviously, such an algorithm aims not to replace a selection committee but, rather, help it rank and select proposals. The method is not specific to any reviewing agency. Actually, it may be applied in any peer-review process requiring reviewer to comment on a proposal (whatever the range of notes). The proposed algorithm is easy to apply but supposes a quantitative rating of proposals by reviewers. This approach may also find application in other contexts such as ranking abstracts submitted to a conference, as was done for the 2010 annual meeting of the French pediatric society [10].

## Supporting Information

Appendix S1 **Giraudeau** ***et al*** **. formula** [**9**] **.**
(DOC)

Appendix S2 **A simulation study to assess the accuracy of the Giraudeau** ***et al.*** **formula (8) in the unbalanced case.**
(DOC)

Appendix S3 **Algorithm code in R language (R Project for Statistical Computing v2.8.1).**
(DOC)
