# Supplementary material for: Peer Review of Grant Applications: A Simple Method to Identify Proposals with Discordant Reviews
Source: PLoS One. 2011 Nov 14;6(11):e27557. doi: 10.1371/journal.pone.0027557 (PMC3215721; doi:10.1371/journal.pone.0027557)
Supplement: Appendix S1 — Giraudeau et al . formula [9] . (DOC) [file pone.0027557.s001.doc]

**Appendix S1: Giraudeau *et al*. formula [9]**

Consider a dataset of *n* proposals, each proposal rated by *p* reviewers. Rho is the intraclass correlation coefficient (ICC) estimated from this dataset. We name $\text{Rho}^{i_{0}}$ as the ICC estimated after proposal *i_0_* has been discarded. Giraudeau *et al.* [9] demonstrated the following result:

$$\text{Rho-Rho}^{i_{0}}=\left(1-\text{Rho}\right)\frac{\frac{n}{n-1}M^{i_{0}}}{nVAR-V^{i_{0}}-\frac{n}{n-1}M^{i_{0}}}-\left(\frac{1}{\left(\text{p-1}\right)}+\text{Rho}\right)\frac{V^{i_{0}}}{nVAR-V^{i_{0}}-\frac{n}{n-1}M^{i_{0}}}$$

where

VAR is the maximum likelihood global variance estimate (i.e., on the whole dataset);

is the squared difference between $m^{i_{0}}$, the rating mean estimate for proposal *i_0_*, and *m*, the global mean estimate;

$V^{i_{0}}$ is the maximum likelihood intra-proposal variance estimate for proposal *i_0_*.

The first term is positive. The more $m^{i_{0}}$ differs from *m*, the higher the term.

The second term is negative. The higher the $V^{i_{0}}$, the higher the term, in absolute value.
