# Supplementary material for: Peer Review of Grant Applications: A Simple Method to Identify Proposals with Discordant Reviews
Source: PLoS One. 2011 Nov 14;6(11):e27557. doi: 10.1371/journal.pone.0027557 (PMC3215721; doi:10.1371/journal.pone.0027557)
Supplement: Appendix S2 — A simulation study to assess the accuracy of the Giraudeau et al. formula (8) in the unbalanced case. (DOC) [file pone.0027557.s002.doc]

**Appendix S2: A simulation study to assess the accuracy**

**of the Giraudeau *et al.* formula (8) in the unbalanced case**

The Giraudeau et al. formula (8) was developed in the balanced case (i.e., with a fixed number of ratings per observation or a fixed number of reviews per proposal in our example). To assess the accuracy of this formula in the unbalanced case (i.e., a variable number of reviews per proposal), we performed a simulation study, according to the following algorithm:

1. Specify *n*, the number of proposals, and *p*, the number of requested reviews
2. Specify *τ*, the overall response rate among reviewers. The mean number of reviews for a proposal is *p τ*
3. Specify *ρ*, the intraclass correlation coefficient (ICC), which defines the level of agreement among reviews
4. For *i_0_* = 1 … *n*:
   - Generate , the number of reviews collected for proposal *i_0_* from a Poisson distribution with mean *p τ*. Consider as the number of reviews received for proposal *i_0_* such that the number of received reviews is ≤ the number of requested reviews
   - Generate data for proposal *i_0_* according to the following model:

where $Y_{i_{0}j}$ is the *j*th review of proposal *i_0_,* $\mu$ is the overall mean, the proposal effects are and the error effects are .

Without loss of generality, we consider that *μ* = 0 and that the overall variance (defined as ) = 1. In this model, the ICC is the proportion of total variance between proposals:

1. Calculate $\hat{\rho}$, the ANOVA estimate of *ρ* (in absence of explicit maximum likelihood estimator when the number of ratings per proposal is variable [8]).
2. For *i_0_* = 1 … *n*:
   - Calculate $\hat{\rho}^{i_{0}}$, the ANOVA estimate of $\rho^{i_{0}}$, the ICC after proposal *i_0_* has been discarded
   - Estimate the two terms of the Giraudeau *et al.* formula (cf Appendix 1), which we name (which depends on the mean estimate for proposal *i_0_*) and (which depends on the maximum likelihood intra-proposal variance estimate for proposal *i_0_*). For term , *p*, the fixed number of ratings per proposal, will be replaced by $p_{0}$, a weighted mean proposal number of rating defined as follows:

where $N=\sum_{i=1}^{n} p_{i}$ is the total number of available ratings

- - Calculate:

which expresses the accuracy of Giraudeau *et al*. formula in the unbalanced case.

Steps 4 to 6 were run 1,000 times and descriptive statistics were estimated from the 1,000**n* observed $D^{i_{0}}$ values.

Simulations considered the following combinations of the fixed parameters: *n* = (20, 50, 100), *p* = (3, 5), *τ* = (0.6, 0.75, 0.9) and *ρ* = (0.5, 0.7, 0.9).

**Results**

Median and inter-quartile ranges of $D^{i_{0}}$ values are in Table S1. The Giraudeau *et al*. formula shows good accuracy in the unbalanced case, with median $D^{i_{0}}$ values close to 0.

**Table S1:** Assessment of Giraudeau *et al*. formula accuracy in the unbalanced case (i.e., when the number of peer-review ratings varies among proposals)

|  |  |  |  | *p*=3 |  | *p*=5 |
| --- | --- | --- | --- | --- | --- | --- |
|  | *τ* | *ρ* |  |  |  |  |
|  | 0.6 | 0.5 |  | -0.040 [-0.087 ; -0.016] |  | -0.031 [-0.059 ; -0.015] |
|  |  | 0.7 |  | -0.029 [-0.065 ; -0.011] |  | -0.024 [-0.045 ; -0.011] |
|  |  | 0.9 |  | -0.011 [-0.026 ; -0.004] |  | -0.009 [-0.019 ; -0.004] |
| *n* = 20 | 0.75 | 0.5 |  | -0.035 [-0.074 ; -0.014] |  | -0.029 [-0.053 ; -0.014] |
|  |  | 0.7 |  | -0.025 [-0.055 ; -0.010] |  | -0.022 [-0.041 ; -0.011] |
|  |  | 0.9 |  | -0.010 [-0.022 ; -0.004] |  | -0.009 [-0.017 ; -0.004] |
|  | 0.9 | 0.5 |  | -0.031 [-0.065 ; -0.013] |  | -0.028 [-0.049 ; -0.015] |
|  |  | 0.7 |  | -0.023 [-0.049 ; -0.010] |  | -0.022 [-0.038 ; -0.011] |
|  |  | 0.9 |  | -0.009 [-0.019 ; -0.003] |  | -0.009 [-0.016 ; -0.004] |
|  |  |  |  |  |  |  |
|  | 0.6 | 0.5 |  | -0.014 [-0.031 ; -0.006] |  | -0.012 [-0.022 ; -0.006] |
|  |  | 0.7 |  | -0.010 [-0.022 ; -0.004] |  | -0.009 [-0.016 ; -0.004] |
|  |  | 0.9 |  | -0.004 [-0.009 ; -0.001] |  | -0.003 [-0.007 ; -0.002] |
| *n* = 50 | 0.75 | 0.5 |  | -0.012 [-0.027 ; -0.005] |  | -0.011 [-0.020 ; -0.005] |
|  |  | 0.7 |  | -0.009 [-0.019 ; -0.003] |  | -0.008 [-0.015 ; -0.004] |
|  |  | 0.9 |  | -0.003 [-0.008 ; -0.001] |  | -0.003 [-0.006 ; -0.002] |
|  | 0.9 | 0.5 |  | -0.011 [-0.024 ; -0.005] |  | -0.011 [-0.019 ; -0.005] |
|  |  | 0.7 |  | -0.008 [-0.018 ; -0.003] |  | -0.008 [-0.014 ; -0.004] |
|  |  | 0.9 |  | -0.003 [-0.007 ; -0.001] |  | -0.003 [-0.006 ; -0.003] |
|  |  |  |  |  |  |  |
|  | 0.6 | 0.5 |  | -0.007 [-0.015 ; -0.003] |  | -0.006 [-0.011 ; -0.003] |
|  |  | 0.7 |  | -0.005 [-0.011 ; -0.002] |  | -0.004 [-0.008 ; -0.002] |
|  |  | 0.9 |  | -0.002 [-0.004 ; -0.001] |  | -0.002 [-0.003 ; -0.001] |
| *n* = 100 | 0.75 | 0.5 |  | -0.006 [-0.013 ; -0.002] |  | -0.005 [-0.010 ; -0.003] |
|  |  | 0.7 |  | -0.004 [-0.009 ; -0.002] |  | -0.004 [-0.007 ; -0.002] |
|  |  | 0.9 |  | -0.002 [-0.004 ; -0.001] |  | -0.002 [-0.003 ; -0.001] |
|  | 0.9 | 0.5 |  | -0.006 [-0.012 ; -0.002] |  | -0.005 [-0.009 ; -0.003] |
|  |  | 0.7 |  | -0.004 [-0.009 ; -0.002] |  | -0.004 [-0.007 ; -0.002] |
|  |  | 0.9 |  | -0.002 [-0.003 ; -0.001] |  | -0.002 [-0.003 ; -0.001] |

Results are expressed as median and inter-quartile ranges of $D^{i_{0}}$ values and derived from 1,000 simulated datasets for each combination (*n*, *p*, *τ*, *ρ*) where *n* is the number of proposals, *p* the number of requested reviews, *τ* the response rate among reviewers and *ρ* the agreement among reviewers.
