# Supplementary material for: Peer Review of Grant Applications: A Simple Method to Identify Proposals with Discordant Reviews
Source: PLoS One. 2011 Nov 14;6(11):e27557. doi: 10.1371/journal.pone.0027557 (PMC3215721; doi:10.1371/journal.pone.0027557)
Supplement: Appendix S3 — Algorithm code in R language (R Project for Statistical Computing v2.8.1). (DOC) [file pone.0027557.s003.doc]

**Appendix S3: Algorithm code in R language (R Project for Statistical Computing v2.8.1)**

#Dataset with one row by proposal and one column for proposal ID number#

#Input the dataset

tab1<-read.csv("the path to the dataset",header=T,sep=";",dec=",")#Here the exmaple is given for a csv dataset#

dim1<-dim(tab1)

nbcol<-dim1[2]

nbrow<-dim1[1]

maxp<-nbcol-1

ntot<-nbrow

tab1<-tab1[order(tab1[,1]),]

#Choice of threshold value for ICC

threshold<-0.7

#Calculation of p, the rating number for each proposal

tab_na<-is.na(tab1[,-c(1)])

p<-(maxp-apply(tab_na, MARGIN=1,FUN=sum))

tab1[,"p"]<-p

#Estimation of mean and sd for each proposal

vect1<-1:nbrow

mat<-as.matrix(tab1[,-c(1,nbcol+1)])

m<-apply(mat,MARGIN=1,FUN=mean,na.rm=T)

sigma<-apply(mat,MARGIN=1,FUN=sd,na.rm=T)

tab2<-data.frame(ID=tab1[,1],Mean=m,SD=sigma)

#Deletion of proposal with only one rating

miss<-NULL

select<-(tab1[,"p"]<2)

temp<-which(select)

miss<-c(miss,temp)

miss<-sort(unique(miss))

suppr<-tab1[miss,]

if (nrow(suppr)>0){

suppr["Order"]<-NA

}

#Create initial dataset

init.table<-tab1[tab1$p>1,]

cci.table<-merge(init.table,tab2,by="ID")

#ICC

#Calculation of n(number of proposals)

k<-dim(cci.table)[1]

#Calculation of the global mean for n*p ratings (p number of ratings per proposal)

mat<-cci.table[,-c(1,maxp+2,maxp+3,maxp+4)]

M<-sum(cci.table[,"p"])

Ybar<-sum(apply(mat,MARGIN=1,FUN=sum,na.rm=T))/M

Mi<-cci.table[,"p"]

Yi<-cci.table[,"Mean"]

Mbar<-sum((Mi**2)/M)

m0=(M-Mbar)/(k-1)

SCb<-sum(Mi*(Yi-Ybar)**2)

SCw<-sum((mat-Yi)**2,na.rm=T)

MSb<-SCb/(k-1)

MSw<-SCw/(M-k)

rho<-(MSb-MSw)/(MSb+(m0-1)*MSw)

#Initiate the identification procedure of proposals to be adjudicated (=with disagreements)

cci.temp<-cci.table

ord<-1

icc<-rho

while (rho<threshold){

#Mi0 calculation

cci.temp$Mi0<-(cci.temp$Mean-Ybar)^2

#Vi0 calculation

cci.temp$Vi0<-(((Mi-1)/Mi)*(cci.temp$SD)^2)

mat2<-cci.temp[,-c(1,maxp+2,maxp+3,maxp+4,maxp+5,maxp+6)]

#Sigma squared calculation

sumdelta<-sum((mat2-Ybar)^2,na.rm=T)

sigmasq<-sumdelta/M

#Second term calculation of Giraudeau et al's formula

cci.temp$T2<-(((1/(Mi-1))+rho)*(cci.temp$Vi0/(k*sigmasq-cci.temp$Vi0-(k/(k-1)*cci.temp$Mi0))))

#Identify proposal ID number(s) which has(ve) the greatest second term value and add it(them) in a suppr dataset

obs1<-(cci.temp[cci.temp$T2==max(cci.temp$T2),])

obs2<-obs1[,seq(1,maxp+2)]

obs2["Order"]<-ord

suppr<-rbind(suppr,obs2)

#Discard this proposal from the initial dataset

cci.temp<-cci.temp[cci.temp$T2<max(cci.temp$T2),]

#Repeat previous steps

k<-dim(cci.temp)[1]

mat<-cci.temp[,-c(1,maxp+2,maxp+3,maxp+4,maxp+5,maxp+6,maxp+7)]

M<-sum(cci.temp[,"p"])

Ybar<-sum(apply(mat,MARGIN=1,FUN=sum,na.rm=T))/M

Mi<-cci.temp[,"p"]

Yi<-cci.temp[,"Mean"]

Mbar<-sum((Mi**2)/M)

m0=(M-Mbar)/(k-1)

SCb<-sum(Mi*(Yi-Ybar)**2)

SCw<-sum((mat-Yi)**2,na.rm=T)

MSb<-SCb/(k-1)

MSw<-SCw/(M-k)

rho<-(MSb-MSw)/(MSb+(m0-1)*MSw)

icc<-c(icc,rho)

ord<-ord+1}

#Create the final dataset which contains all proposals to be adjudicated

discuss<-merge(suppr,tab2,by="ID")

discuss<-discuss[order(discuss[,"Order"])]

icc

discuss
